# Neoadjuvant Radiotherapy vs Up-Front Surgery for Resectable Locally Advanced Rectal Cancer

**DOI:** 10.1001/jamanetworkopen.2025.9049

**Published:** 2025-05-07

**Authors:** Po-Chuan Chen, Avery Shuei-He Yang, Alessandro Fichera, Mu-Hung Tsai, Yuan-Hua Wu, Yu-Min Yeh, Yu Shyr, Edward Chia-Cheng Lai, Chao-Han Lai

**Affiliations:** 1 Department of Surgery, National Cheng Kung University Hospital, College of Medicine, National Cheng Kung University, Tainan, Taiwan; 2 School of Pharmacy, Institute of Clinical Pharmacy and Pharmaceutical Sciences, College of Medicine, National Cheng Kung University, Tainan, Taiwan; 3 Population Health Data Center, National Cheng Kung University, Tainan, Taiwan; 4 Division of Colon and Rectal Surgery, Department of Surgery, Weill Cornell Medicine, New York, New York; 5 Department of Radiation Oncology, National Cheng Kung University Hospital, College of Medicine, National Cheng Kung University, Tainan, Taiwan; 6 Department of Oncology, National Cheng Kung University Hospital, College of Medicine, National Cheng Kung University, Tainan, Taiwan; 7 Department of Biostatistics, Vanderbilt University Medical Center, Nashville, Tennessee; 8 Department of Biochemistry and Molecular Biology, College of Medicine, National Cheng Kung University, Tainan, Taiwan

## Abstract

**Importance:**

Guidelines for resectable locally advanced rectal cancer (LARC) advocate for neoadjuvant radiotherapy (NRT) followed by surgery as the standard approach. However, recent trials have reported no oncological benefits of NRT-based therapy for middle or lower rectal cancer, raising the question of whether NRT followed by surgery remains the optimal treatment approach for resectable LARC overall.

**Objective:**

To compare the outcomes of NRT followed by surgery vs up-front surgery for resectable LARC.

**Design, Setting, and Participants:**

This cohort study, using a target trial emulation framework with nationwide registries in Taiwan, included patients undergoing curative resection for resectable LARC (cT1-2N1-2, cT3Nany) between January 1, 2014, and December 31, 2017, with follow-up until December 31, 2020. Data were analyzed from January 1, 2024, to February 15, 2025.

**Exposure:**

NRT.

**Main Outcomes and Measures:**

The primary outcomes were overall survival (OS) and local recurrence (LR). The secondary outcome was intraoperative diverting stoma outcomes.

**Results:**

A total of 4099 patients were analyzed, including 1436 patients undergoing NRT followed by surgery (median [IQR] age, 62.0 [53.0-71.0] years; 1036 [72.1%] male) and 2663 patients undergoing up-front surgery (median [IQR] age, 65.0 [56.0-74.0] years; 1626 [61.1%] male). NRT followed by surgery, compared with up-front surgery, was associated with higher 3-year OS rates (88.5% vs 85.2%; hazard ratio [HR], 0.74; 95% CI, 0.59-0.92) but higher permanent diverting stoma rates (20.6% vs 11.1%; relative risk [RR], 1.91; 95% CI, 1.62-2.25); LR rates were not significantly different (5.7% vs 6.6%; HR, 0.78; 95% CI, 0.55-1.11). Subgroup analysis revealed that compared with up-front surgery, NRT followed by surgery was associated with improved outcomes in middle or lower rectal cancer but not upper rectal cancer (OS: HR, 1.54; 95% CI, 0.82-2.90; LR: HR, 1.08; 95% CI, 0.23-5.00). NRT followed by surgery was associated with significantly increased risks of permanent diverting stomas across different tumor heights, particularly in upper rectal cancer (RR, 3.54; 95% CI, 1.44-8.69).

**Conclusions and Relevance:**

In this cohort study of nationwide registries in Taiwan, NRT followed by surgery was associated with improved oncological outcomes for overall resectable LARC, with excessive diverting stoma nonreversal as the trade-off. However, the benefits of NRT were not observed for upper rectal cancer. These findings raise concerns about potential harm from NRT and advise caution when performing NRT for upper rectal cancer.

## Key Points

**Question:**

What are the outcomes associated with neoadjuvant radiotherapy (NRT) followed by surgery compared with up-front surgery for patients with resectable locally advanced rectal cancer (LARC)?

**Findings:**

This cohort study including 4099 patients from nationwide registries found that compared with up-front surgery, NRT before surgery was associated with improved oncological outcomes at the cost of increased risk of diverting stoma nonreversal. Subgroup analysis found these benefits for middle or lower LARC but not upper LARC.

**Meaning:**

These findings suggest that the trade-off between NRT use and diverting stoma nonreversal may not be justified for upper rectal cancer, emphasizing the importance of considering tumor height when treating resectable LARC.

## Teaser

This cohort study compared outcomes for neoadjuvant radiotherapy followed by surgery vs up-front surgery for patients with resectable locally advanced rectal cancer.

## Introduction

Colorectal cancer poses a significant threat to global health, ranking as the third most commonly diagnosed cancer and second leading cause of cancer-related deaths worldwide.^1^ Compared with colon cancer, surgical resection of rectal cancer has historically been considered technically challenging because of the confinement of the bony pelvis and its anatomical proximity to the anal sphincter and other pelvic organs.^2^ A pooled analysis of 14 randomized clinical trials (RCTs) conducted by the end of the 20th century showed that compared with up-front surgery, radiotherapy as neoadjuvant treatment followed by surgery reduced local recurrence (LR) and improved survival for patients with resectable locally advanced rectal cancer (LARC).^3^ While the Dutch^4^ and German^5^ trials in the early 2000s showed no difference in survival between patients who received neoadjuvant radiotherapy (NRT) followed by surgery and those who received up-front surgery, both studies revealed a lower LR rate under the NRT-based strategy. The NSABP R-03 trial^6^ found that neoadjuvant chemoradiotherapy did not statistically significantly improve overall survival (OS) nor did it reduce LR. However, the international, multicenter MRC CR07 trial^7^ demonstrated the benefit of NRT on local control. These results underpin the NRT-based approach for resectable stage II to III LARC recommended in most guidelines,^8,9,10,11^ despite potential overtreatment recognized in selected patients.^12,13,14^

Landmark RCTs revealed the effect of NRT followed by surgery in improving oncological outcomes, yet only a few of these groups reported the rates and consequences of stoma creation.^15,16^ A secondary analysis^16^ of the trial by Kapiteijn et al^4^ found that NRT was associated with a decreased likelihood of stoma reversal for secondary stomas (ie, stomas created during a second procedure after oncological resection).^16^ Creating a diverting stoma to reduce the risk of symptomatic anastomotic leak has been an essential consideration in surgical decisions for sphincter-preserving surgery.^17,18^ The incidence of intraoperative diverting stoma creation following NRT can be strikingly high, exceeding 90%.^19,20,21^ Unfortunately, a substantial proportion of these diverting stomas initially intended to be temporary ultimately become permanent,^22^ and NRT has been identified as a risk factor for permanent diverting stomas.^23^ Patients with a stoma, particularly a permanent stoma, have significantly worse quality of life than patients without stoma.^24,25^ In addition, the nonreversal of diverting stomas contradicts its original intent and, to some extent, undermines the significance of sphincter preservation. The optimal treatment strategy for resectable LARC requires balancing oncological outcomes with quality of life.^26^ This issue is particularly crucial when potential overtreatment resulting from the routine practice of NRT is a concern.

Advancements in surgical techniques, along with the use of magnetic resonance imaging (MRI) for preoperative decision-making and collaboration among multidisciplinary team members, have reduced rates of involved surgical margins and LR after surgery for LARC over time.^27^ More recently, the PROSPECT trial has shown the noninferiority of neoadjuvant chemotherapy with fluorouracil, leucovorin, and oxaliplatin to the prevailing standard of neoadjuvant chemoradiotherapy.^28^ Also, 3 consecutive RCTs have demonstrated no oncological benefits of NRT-based treatment for middle or lower rectal cancer.^29,30,31^ These results reasonably raise the question of whether NRT-surgery remains the optimal treatment approach for overall resectable LARC today. A large-scale clinical study has the potential to assess whether the results seen in RCTs would be generalizable to clinical patient populations.^32^ We conducted a target trial emulation analysis,^33^ obtaining data from linked nationwide databases in Taiwan, to evaluate OS, LR, and diverting stoma outcomes among patients who underwent NRT followed by surgery compared with those who underwent up-front surgery for resectable LARC.

## Methods

This cohort study was approved by the institutional review board of National Cheng Kung University Hospital. Informed consent was waived due to the retrospective collection of anonymized data. This study followed the Strengthening the Reporting of Observational Studies in Epidemiology (STROBE) reporting guideline.

### Specification of the Target Trial

Data for this target trial were collected from the Taiwan Cancer Registry Database (TCRD) and the Taiwan National Health Insurance Database (NHIRD). Both databases are notable for their high data accuracy and completeness.^34,35^ The TCRD, derived from the government-supported nationwide cancer registry system established in 1996, collected data from 214 hospitals with 50 beds or more mandated to report cancer cases.^34^ Detailed cancer information (eg, tumor stage and histology) was obtained from the TCRD. The NHIRD is a nationwide claims database covering nearly all the population (approximately 99%) in Taiwan,^35^ providing comprehensive information on diagnoses, procedures, and medications.

eFigure 1 in Supplement 1 summarizes patient selection. The target trial included adult patients aged 20 years or older diagnosed with clinically resectable LARC (using the diagnosis codes c19.9 and c20.9 defined by the *International Classification of Diseases for Oncology, Third Edition* [*ICD-O-3*], and clinical stages cT1-2N1-2M0 and cT3NanyM0) between January 1, 2014, and December 31, 2017. Patients who had nonadenocarcinoma pathologies, did not undergo curative resection, lacked information about surgery, had treatment initiated more than 6 weeks after diagnosis, or underwent stoma creation before treatment initiation were excluded. Eligible patients were randomly allocated to 1 of 2 treatment groups: (1) NRT followed by surgery (ie, patients undergoing preoperative radiotherapy before curative resection) or (2) up-front surgery (ie, no radiotherapy before curative resection) after the diagnosis of LARC. Comorbidities listed in the Charlson Comorbidity Index and postoperative complications were identified using the *International Classification of Diseases, Ninth Revision, Clinical Modification *(*ICD-9-CM*) and *International Statistical Classification of Diseases, Tenth Revision, Clinical Modification *(*ICD-10-CM*), codes (eTable 2 in Supplement 1). Other tumor-related clinical parameters (eg, pathological stage and surgical margin status) were also recorded.

### Emulation of the Target Trial

Target trial emulation is a framework designed to apply the fundamental principles of clinical trials to enhance the rigor of observational studies.^36,37^ Incorporating this framework into a conventional retrospective propensity score–based cohort study helps structure the study using a standard design that improves causal inference. eTable 1 in Supplement 1 summarizes the key protocol components of the trial emulation process, including aim, eligibility criteria, treatment assignment, and outcomes. To emulate the random assignment, we implemented propensity score fine stratification with average treatment effect among the treated population weighting.^38^ We created 100 equally sized propensity score strata based on the propensity score distribution of whole study population. Weights for each individual were subsequently calculated based on the patient number among each stratum to account for stratum membership.^39,40^ Specifically, the treated patients (ie, patients assigned to the NRT followed by surgery group) received a weight of 1, while the patients receiving up-front surgery were weighted by the relative ratio of patients number within each stratum as weight = ([N_NRT-surgery in stratum i_ ⁄ N_total NRT-surgery_] / [N_upfrount surgery in stratum i_ / N_total up-front surgery_]), where *NRT-surgery* indicates the group that received NRT followed by surgery. This approach minimizes extreme weights and increases the precision of the estimator, which is a common issue when using propensity score weighting, particularly when the exposure is infrequent.

The index date was the date of treatment assignment (ie, first NRT for patients with NRT followed by surgery and surgery for patients with up-front surgery). All patients were followed-up from the index date until death or December 31, 2020, whichever came first. The primary outcomes of interest were OS and LR. The secondary outcome was intraoperative diverting stoma outcomes, specifically unreversed (permanent) diverting stomas 3 years after surgery.

### Statistical Analysis

Data are presented as number (percentage) or median (IQR) as indicated. An absolute standardized mean difference greater than 0.10 between groups was considered significantly different. The propensity score was calculated based on the age, sex, body mass index, Charlson Comorbidity Index, clinical stage, tumor height (upper, >10 cm; middle, 5-10 cm; and lower, <5 cm from the anal verge),^5^ surgical approach (open or laparoscopic/robotic), level of hospital where treatment was conducted, and calendar year. Hazard ratios (HRs) of OS and LR (primary outcomes) were analyzed using the Cox proportional hazards model, for which the causal estimates of interest were based on an as-started approach, analogous to the intention-to-treat approach, and survivals between groups were presented via Kaplan-Meier curves. Stoma outcomes were recorded every 6 months during follow-up after surgery. The relative risks (RRs) of unreversed diverting stomas (secondary outcome) were estimated using Poisson regression. Subgroup analysis of the weighted population was conducted to examine the associations of NRT with OS, LR, and diverting stoma outcomes according to tumor height. Adjustments for unbalanced covariates were performed to ensure balanced characteristics among subgroups. Multivariate Cox regression analysis was performed as a sensitivity analysis to validate the results of primary outcomes. For comparison with RCTs published previously,^4,5,6,7,29,30,31,41,42,43,44,45^ we applied the rectangular function in the ImageJ software version 1.54g (National Institutes of Health) or JAVA version 1.8.0_345 (64-bit) (Oracle) to estimate the 3-year OS and LR rates through the Kaplan-Meier curves if the data were not specifically reported.^46^ All analyses were conducted with SAS software version 9.4 (SAS Institute) and Excel 2016 (Microsoft). A 2-sided *P* < .05 was considered statistically significant. Data were analyzed from January 1, 2024, to February 15, 2025.

## Results

### Baseline Characteristics and Treatments

A total of 4099 patients were eligible for analysis, including 1436 patients who underwent NRT followed by surgery (median [IQR] age, 62.0 [53.0-71.0] years; 1036 [72.1%] male) and 2663 patients who underwent up-front surgery (median [IQR] age [IQR], 65.0 [56.0-74.0] years; 1626 [61.1%] male) (Table 1). Compared with patients with up-front surgery, patients with NRT followed by surgery were younger and more likely to be male, have advanced N-stage disease, have lower rectal cancer, undergo laparoscopic/robotic surgery, and receive treatment at tertiary referral centers.

**Table 1.** Baseline Characteristics of Patients With Resectable LARC Receiving Up-Front Surgery vs NRT Followed by Surgery Before and After Propensity Score Fine Stratification

Variables^a^	Before propensity score fine stratification			After propensity score fine stratification		
	Patients, No. (%)		SMD	Patients, No. (%)		SMD
	NRT-surgery (n = 1436)	Up-front surgery n = 2663)		NRT-surgery (n = 1308)	Up-front surgery (n = 2484)	
Age, median (IQR), y	62.0 (53.0-71.0)	65.0 (56.0-74.0)	−0.31	62.0 (53.0-71.0)	62.0 (53.0-71.0)	−0.06
Sex						
Male	1036 (72.1)	1626 (61.1)	0.24	932 (71.3)	1796 (72.3)	−0.02
Female	400 (27.9)	1037 (38.9)		376 (28.7)	688 (27.7)	
BMI, median (IQR)	24.1 (21.8-26.5)	23.8 (21.4-26.2 )	0.06	24.1 (21.7-26.5)	23.9 (21.6-26.2)	0.00
Charlson comorbidity index, median (IQR)	3 (2-4)	4 (2-8)	0.06	3 (2-4)	3 (2-4)	0.00
Clinical stage						
cT1	160 (11.1)^a^	13 (0.5)	−0.06	148 (0.20)^a^	3 (0.1)	0.01
cT2		284 (10.7)	0.01		294 (11.8)	−0.02
cT3	1276 (88.9)	2366 (88.9)	0.00	1160 (88.7)	2187 (88.1)	0.02
cN0	318 (22.1)	1028 (38.6)	−0.36	292 (22.3)	559 (22.5)	0.00
cN1	568 (39.6)	1132 (42.5)	−0.06	532 (40.7)	959 (38.6)	0.04
cN2	550 (38.3)	503 (18.9)	0.44	484 (37.0)	966 (38.9)	−0.04
Tumor height						
Upper rectal cancer	78 (5.4)	799 (30)	−0.68	72 (5.5)	144 (5.8)	−0.01
Middle rectal cancer	550 (38.3)	1279 (48)	−0.20	519 (39.7)	995 (40.1)	−0.01
Lower rectal cancer	808 (56.3)	585 (22)	0.75	717 (54.8)	1345 (54.2)	0.01
Surgical approach						
Open surgery	609 (42.4)	1362 (51.2)	−0.18	567 (43.4)	1049 (42.2)	0.02
Laparoscopic/robotic surgery	827 (57.6)	1301 (48.9)	0.18	741 (56.7)	1434 (57.8)	−0.02
Tertiary referral center	759 (52.9)	840 (31.5)	0.44	653 (49.9)	1224 (49.3)	0.01
Year						
2014	415 (28.9)	822 (30.9)	−0.04	369 (28.2)	745 (30.0)	−0.04
2015	401 (27.9)	596 (22.4)	0.13	354 (27.1)	651 (26.2))	0.02
2016	343 (23.9)	579 (21.7)	0.05	321 (24.5)	606 (24.4)	0.00
2017	277 (19.3)	666 (25.0)	−0.14	264 (20.2)	483 (19.4)	0.02

Abbreviations: BMI, body mass index (calculated as weight in kilograms divided by height in meters squared); LARC, locally advanced rectal cancer; NRT, neoadjuvant radiotherapy; SMD, standardized mean difference.

^a^ The patient numbers of cT1 and cT2 cannot be reported separately in accordance with privacy policy of the Taiwan National Health Insurance Database.

After propensity score fine stratification, 3792 patients (1308 patients in the NRT followed by surgery group; 2484 patients in the up-front surgery group) were included in this emulated target trial. The groups were balanced regarding the distribution of the variables considered. Among patients undergoing NRT followed by surgery, 1077 patients (82.3%) received long-course radiotherapy, and 1028 patients (78.6%) received concurrent 5-fluorouracil–based chemotherapy (eTable 3 in Supplement 1). After surgery, patients with NRT prior to surgery were less likely to receive adjuvant radiotherapy but more likely to receive 5-fluorouracil–based adjuvant chemotherapy than patients with up-front surgery.

### Perioperative Outcomes

Pathological analysis found that NRT was associated with the downstaging of LARC (eTable 4 in Supplement 1). Notably, the involved surgical margin rates were not significantly different between patients with NRT prior to surgery and those with up-front surgery (3.1% vs 4.9%; standardized mean difference, 0.09). Regarding postoperative complications, deep vein thrombosis occurred more frequently among patients with NRT before surgery than those with up-front surgery, and the incidences of other complications were comparable between groups.

### Primary and Secondary Outcomes

**Figure 1.**
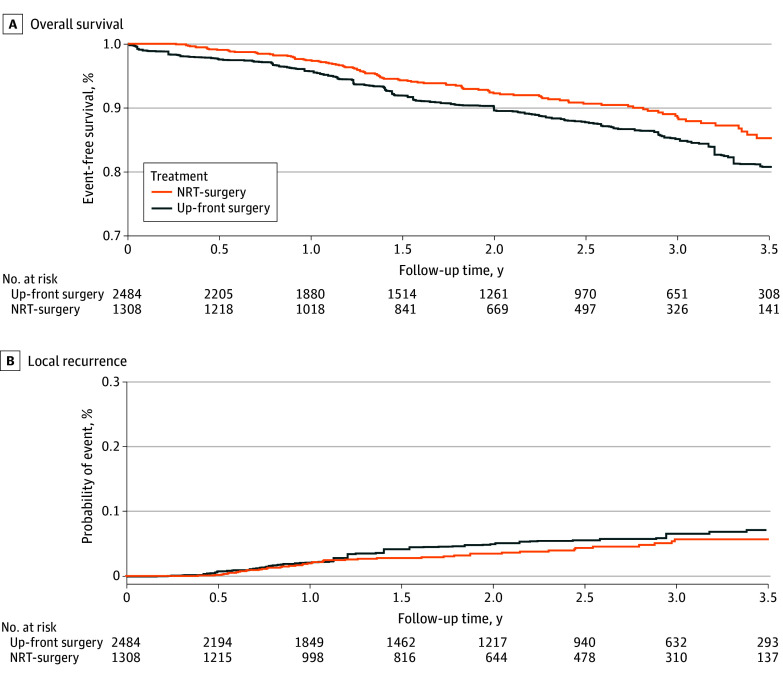
Kaplan-Meier Estimates of Overall Survival and Local Recurrence for Patients With Neoadjuvant Radiotherapy (NRT) Followed by Surgery or Up-Front Surgery

All 3792 patients were followed up for at least 3 years. Compared with up-front surgery, NRT followed by surgery was associated with higher 3-year OS rates (85.2% vs 88.5%; HR, 0.74; 95% CI, 0.59-0.92) (Figure 1A), indicating a survival benefit for NRT. Compared with up-front surgery, NRT followed by surgery was not associated with significantly different LR rates (6.6% vs 5.7%; HR, 0.78; 95% CI, 0.55-1.11) (Figure 1B).

**Figure 2.**
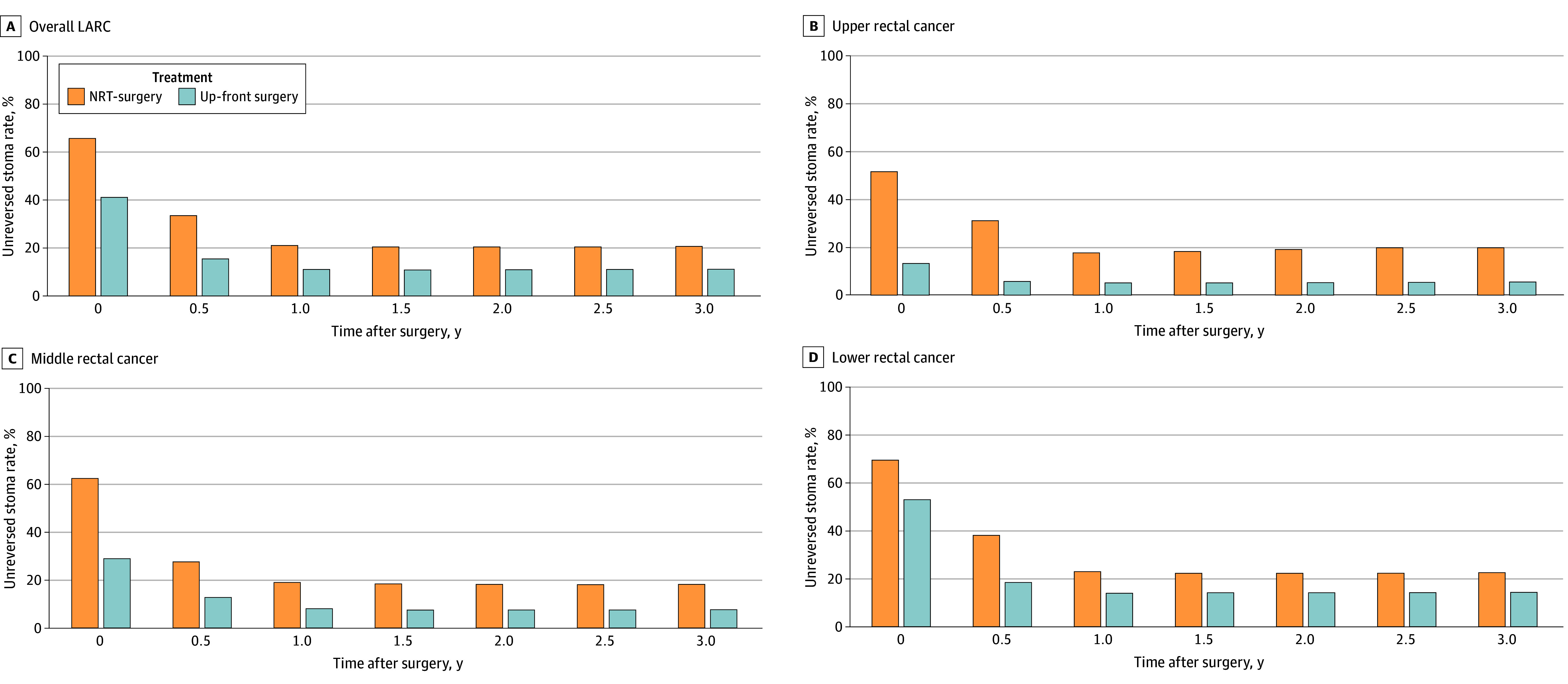
Unreversed Diverting Stoma Rates for Overall Locally Advanced Rectal Cancer (LARC) and by LARC Height for Patients With Neoadjuvant Radiotherapy Followed by Surgery or Up-Front Surgery

During surgery, 1875 patients (49.4%) underwent diverting stoma creation. Compared with up-front surgery, NRT followed by surgery was associated with higher rates of diverting stoma creation (41.0% vs 65.5%; RR, 1.60; 95% CI, 1.46-1.75) (Figure 2A). While most stomas (1343 stomas [71.6%]) were closed within 1 year, the higher risk for unreversed stomas after NRT before surgery persisted. At 3 years, 498 stomas (26.6%) remained unreversed, indicating that 13.1% of all patients had a permanent diverting stoma. Compared with up-front surgery, NRT before surgery was associated with higher permanent diverting stoma rates (11.1% vs 20.6%; RR, 1.91; 95% CI, 1.62-2.25).

### Tumor Height

Subgroup analysis was conducted according to tumor height (Table 2). Compared with up-front surgery, NRT followed by surgery was not associated with significantly increased OS (HR, 0.70; 95% CI, 0.48-1.02) in middle rectal cancer but was associated with significantly increased OS (HR, 0.66; 95% CI, 0.46-0.96) and reduced LR (HR, 0.53; 95% CI, 0.30-0.95) in lower rectal cancer. However, NRT followed by surgery was not associated with improved OS (HR, 1.54; 95% CI, 0.82-2.90) or LR (HR, 1.08; 95% CI, 0.23-5.00) in upper rectal cancer.

**Table 2.** Risks of Overall Survival and Local Recurrence at 3 Years After NRT Followed by Surgery vs Up-Front Surgery for Overall Resectable LARC and According to Different Tumor Heights

Primary outcome	% (95% CI)		HR (95% CI)^a^
	NRT-surgery	Up-front surgery	
**Overall survival**			
Overall	88.5 (86.0-90.7)	85.2 (80.3-89.0)	0.74 (0.59-0.92)^a^
Upper rectal cancer	77.5 (62.4-87.1)	81.0 (74.4-86.0)	1.54 (0.82-2.90)
Middle rectal cancer	90.3 (86.5-93.1)	84.5 (79.9-88.2)	0.70 (0.48-1.02)
Lower rectal cancer	88.2 (84.2-91.2)	86.3 (77.5-91.8)	0.66 (0.46-0.96)
**Local recurrence**			
Overall	5.7 (4.1-7.8)	6.6 (4.2-10.4)	0.78 (0.55-1.11)
Upper rectal cancer	4.1 (1.0-16.2)	3.8 (1.8-8.0)	1.08 (0.23-5.00)
Middle rectal cancer	5.3 (3.2-9.0)	5.4 (3.5-8.3)	1.13 (0.60-2.14)
Lower rectal cancer	5.9 (3.9-8.9)	7.8 (3.6-16.8)	0.53 (0.30-0.95)^a^

Abbreviations: HR, hazard ratio; LARC, locally advanced rectal cancer; NRT, neoadjuvant radiotherapy.

^a^ Signifies statistical significance (*P* < .05).

Patients undergoing NRT prior to surgery were more likely to have diverting stomas created than patients undergoing up-front surgery across different heights (Figure 2B-D). Also, both groups were more likely to have stomas created for lower-lying tumors. Notably, the risk of diverting stoma creation comparing NRT prior to surgery vs up-front surgery was highest in upper rectal cancer (RR, 3.61; 95% CI, 2.03-6.43) and lowest in lower rectal cancer (RR, 1.31; 95% CI, 1.17-1.47). Also, the risk of permanent diverting stomas at 3 years was highest in upper rectal cancer (RR, 3.54; 95% CI, 1.44-8.69) and lowest in lower rectal cancer (RR, 1.62; 95% CI, 1.33-1.98).

### Sensitivity Analysis for Primary Outcomes

eTable 5 in Supplement 1 shows the HRs of OS and LR obtained from multivariate Cox regression analysis of the overall 4099 patients with resectable LARC. The results were similar to those obtained from the emulated target trial framework (Table 2). Also, examination of the interaction between tumor height and treatment effect revealed a significant interaction for OS although not for LR.

## Discussion

Using nationwide registries, this cohort study used a target trial emulation framework for head-to-head comparisons of the outcomes associated with NRT followed by surgery vs up-front surgery for resectable LARC overall. Compared with up-front surgery, NRT followed by surgery was associated with improved oncological outcomes for patients with resectable LARC. Subgroup analysis revealed such benefits for middle or lower but not upper rectal cancer. In addition, NRT prior to surgery was associated with an increased risk of diverting stoma creation, with excessive stomas remaining for at least 3 years. The higher risks of diverting stoma creation and nonreversal associated with NRT prior to surgery were observed across different tumor heights, and the risk magnitudes were most pronounced in upper rectal cancer. These findings underscore the importance of considering tumor height when performing NRT for LARC.

Milestone RCTs have established the advantages of NRT for resectable LARC.^4,5,6,7,45^ The MERCURY,^13^ QuikSilver,^12^ and OCUM^14^ studies used high-resolution MRI assessment of circumferential resection margin and explored MRI criteria to identify rectal cancers with good prognosis eligible for up-front surgery.^12,13,14^ With the improvement in quality of rectal cancer surgery, a far greater number of patients now need to be treated with NRT to prevent 1 LR.^47^ The PROSPECT trial^28^ demonstrated that neoadjuvant chemotherapy with fluorouracil, leucovorin, and oxaliplatin can be used in lieu of chemoradiotherapy. Our study, along with these efforts, supports more selective use of NRT for resectable LARC in an era of multidisciplinary approaches and personalized treatment for rectal cancer.

RCTs exploring the effect of NRT on LARC at different tumor heights have shown highly inconsistent results,^4,7,30,48,49,50,51^ which cannot fully support guideline statements.^11,52^ For example, despite having overlapping patient populations, the Stockholm II trial^48^ and Swedish Rectal Cancer Trial^50^ reported contradictory results. Results from MERCURY,^13^ QuikSilver,^12^ and OCUM^14^ studies suggested tumor height as an important consideration for NRT omission. Pathological and radiological evidence support the concept that lower lesions may benefit more from NRT,^53,54^ which facilitates more effective oncological resection. eFigure 2 in Supplement 1 summarizes 3-year oncological outcomes reported in the trials comparing NRT followed by surgery with up-front surgery for resectable LARC. Although NRT still provides oncological benefits, the contribution margin has been steadily narrowing. Improved surgical quality diminishes the advantages of NRT for upper rectal cancer.

In line with high incidence rates of diverting stomas created in patients undergoing NRT prior to surgery,^19,20,21^ a Swedish Colorectal Cancer Registry study^23^ identified NRT as a risk factor for permanent diverting stomas. These observations reflect the apprehensions of surgeons regarding the risk of symptomatic anastomotic leak and the consequences following NRT exposure. Unsurprisingly, NRT and anastomotic and tumor height are considered key factors in intraoperative decision-making for diverting stoma creation,^55,56^ as both are risk factors for anastomotic leak.^17,18^ In this study, patients undergoing NRT prior to surgery were more likely to undergo diverting stoma creation, with more than 25% of these stomas remaining for at least 3 years. Interestingly, the risks of diverting stoma creation and permanent diverting stomas in patients with upper rectal cancer were substantially higher than in patients with lower rectal cancer, despite the lower expected risk of anastomotic leak for upper rectal cancer.^17,18^ For upper rectal cancer, no oncological benefit was observed following NRT, which instead was associated with more diverting stomas, stoma reversal–related procedures and complications, and permanent stomas if left unreversed.

A long-standing controversy exists regarding the optimal approach for upper rectal cancer.^57^ Despite controversial evidence,^4,7,30,48,49,50,51^ the ESMO guidelines^11^ recommend omitting NRT for patients with upper rectal cancers (>12 cm from the anal verge), as opposed to those with middle or lower rectal cancer. In the National Comprehensive Cancer Network guidelines,^8^ advanced therapeutic modalities (eg, neoadjuvant chemotherapy and immunotherapy)^28,58^ are promising alternatives to NRT-based treatment. Recently, total neoadjuvant therapy has gained increasing enthusiasm due to its potential for organ preservation through nonoperative management (ie, watch and wait approach) in patients achieving complete clinical response. However, several potential concerns exist, such as overtreatment, unnecessary chemotherapy toxic effects, and lack of standardized sequencing strategies.^59^ More importantly, total neoadjuvant therapy is associated with suboptimal surgical quality and potentially delays surgery in nonresponders.^60,61^ These considerations are particularly noteworthy for patients with upper rectal cancer, given the potential overtreatment and harm of NRT shown in our study.

### Limitations

We acknowledge several limitations to this study. First, the study might be subject to indication bias (ie, patients with more invasive features beyond the scope of the TNM system might be more likely to undergo NRT) and residual confounding factors (eg, intraoperative details) because of nonrandom allocation through retrospective registry-based data.^62^ Nevertheless, we attempted to mitigate these issues by considering as many potential confounders (eg, tumor stage and hospital level) as possible. A sensitivity analysis using multivariate Cox regression analysis with adjustment for the variables used in the propensity score calculations yielded consistent results. Second, the measurement of tumor height may differ according to the modality used (eg, MRI or sigmoidoscopy).^63^ Although tumor height was a mandatory variable in the TCRD during the study period, information regarding measuring modalities was unavailable. Third, we acknowledge the time gap between NRT and surgery. Our study design aligns with relevant clinical trials and regimens, suggesting minimal impact of this lag time. Additionally, despite the well-maintained data quality of the nationwide databases,^34,35^ a relatively small percentage of missing data or miscoded diagnoses is inherent in registry-based studies. These coding errors are likely random and should not significantly impact the study findings.

## Conclusions

In this cohort study of nationwide registries in Taiwan, NRT prior to surgery was associated with improved outcomes for patients with resectable LARC overall compared with up-front surgery. However, these benefits came at the cost of increased risk of diverting stoma creation and nonreversal. Possibly reflecting quality improvement in surgery in recent years, the oncological benefits of NRT were observed only for middle or lower rectal cancer. Thus, for upper rectal cancer, the trade-off between using NRT to pursue better oncological outcomes and creating diverting stomas to lower the risk of symptomatic leak may not be justified. These findings raise concerns about the potential overtreatment and harm of NRT, emphasizing the importance of considering tumor height when treating resectable LARC.

## Notes

Supplement 1. **eTable 1.** The Target Trial Table **eTable 2.** *International Classification of Diseases, Ninth Revision, Clinical Modification (ICD-9-CM) *and *International Statistical Classification of Diseases, Tenth Revision, Clinical Modification (ICD 10-CM)* **eTable 3.** Neoadjuvant/Adjuvant Therapies of Patients With Resectable LARC After Propensity Score Fine Stratification **eTable 4.** Perioperative Outcomes of Patients With Resectable LARC After Propensity Score Fine Stratification **eTable 5.** Risks of Overall Survival and Local Recurrence at 3 Years Following NRT Followed by Surgery Versus Up-Front Surgery for Overall Resectable LARC and According to Different Tumor Heights Estimated Using Multivariate Cox Regression **eFigure 1.** Patient Selection **eFigure 2.** Bubble Charts

Supplement 2.
Data Sharing Statement
