# Supplementary material for: Neoadjuvant Radiotherapy vs Up-Front Surgery for Resectable Locally Advanced Rectal Cancer
Source: JAMA Netw Open. 2025 May 7;8(5):e259049. doi: 10.1001/jamanetworkopen.2025.9049 (PMC12059978; doi:10.1001/jamanetworkopen.2025.9049)
Supplement: Supplement 1. — eTable 1. The Target Trial Table eTable 2. International Classification of Diseases, Ninth Revision, Clinical Modification (ICD-9-CM) and International Statistical Classification of Diseases, Tenth Revision, Clinical Modification (ICD 10-CM) eTable 3. Neoadjuvant/Adjuvant Therapies of Patients With Resectable LARC After Propensity Score Fine Stratification eTable 4. Perioperative Outcomes of Patients With Resectable LARC After Propensity Score Fine Stratification eTable 5. Risks of Overall Survival and Local Recurrence at 3 Years Following NRT Followed by Surgery Versus Up-Front Surgery for Overall Resectable LARC and According to Different Tumor Heights Estimated Using Multivariate Cox Regression eFigure 1. Patient Selection eFigure 2. Bubble Charts [file jamanetwopen-e259049-s001.pdf]

Supplemental Online Content

Chen PC, Yang ASH, Fichera A, et al. Neoadjuvant radiotherapy vs up-front surgery for resectable locally advanced rectal cancer. *JAMA Netw Open*. 2025;8(5):e259049. doi:10.1001/jamanetworkopen.2025.9049

eTable 1. The Target Trial Table

eTable 2. *International Classification of Diseases, Ninth Revision, Clinical Modification (ICD-9-CM)* and *International Statistical Classification of Diseases, Tenth Revision, Clinical Modification (ICD 10-CM)*

eTable 3. Neoadjuvant/Adjuvant Therapies of Patients With Resectable LARC After Propensity Score Fine Stratification

eTable 4. Perioperative Outcomes of Patients With Resectable LARC After Propensity Score Fine Stratification

eTable 5. Risks of Overall Survival and Local Recurrence at 3 Years Following NRT Followed by Surgery Versus Up-Front Surgery for Overall Resectable LARC and According to Different Tumor Heights Estimated Using Multivariate Cox Regression

eFigure 1. Patient Selection

eFigure 2. Bubble Charts

This supplemental material has been provided by the authors to give readers additional information about their work.

© 2025 Chen PC et al. *JAMA Network Open*.

eTable 1. The target trial table.

Component	Target trial	Target trial emulation using real-world data
**Aim**	1. To compare the oncological outcomes between neoadjuvant radiotherapy (NRT) followed by surgery versus upfront surgery.<br>2. To compare the diverting stoma outcomes between NRT followed by surgery versus upfront surgery.	Same as the target trial
**Eligibility criteria**	**Inclusion:**<br>● Patients aged 20 years and older.<br>● Patients diagnosed with cT1-2N1-2M0 and cT3NanyM0 locally advanced rectal cancer (LARC) between 2014 and 2017.<br><br>**Exclusion:**<br>● Non-adenocarcinoma rectal cancer.<br>● Not undergoing curative resection.<br>● Treatment started over 6 weeks after diagnosis.<br>● Undergoing diverting stoma, abdominoperineal resection, or Hartmann's operation before treatment initiation.	Same as the target trial<br>Patients without records of surgery or information on surgical procedures after LARC diagnosis were additionally excluded.
**Treatment strategy**	1. Patients who received NRT followed by surgery.<br>2. Patients who received upfront surgery.	Same as the target trial
**Treatment assignment**	Eligible patients were randomly allocated to the NRT followed by surgery or upfront surgery group.	The propensity score method with fine stratification weighting generates a study population with balanced characteristics and similar probabilities of treatment assignment.
**Outcomes**	**Primary:**<br>● Overall survival (OS) rate<br>● Local recurrence (LR) rate<br><br>**Secondary:**<br>● Unreversed diverting stoma rate	Same as the target trial
**Follow-up**	From the index date to December 31, 2020, or to the occurrence of outcome events or death	Same as for the target trials
**Causal contrasts**	Intention-to-treat analysis	As-treated analysis, an analog of intention-to-treat
**Statistical analysis**	● For the primary outcomes (i.e., OS and LR): the Cox	Same as the target trial

© 2025 Chen PC et al. *JAMA Network Open*.

	proportional hazard model was implemented to estimate the hazard ratio with 95% confidence interval<br>• For the secondary outcome (i.e., unreversed diverting stoma rate): the Poission regression model was implemented to estimate the risk ratio with 95% confidence interval	

© 2025 Chen PC et al. *JAMA Network Open*.

eTable 2. International Classification of Diseases, Ninth Revision, Clinical Modification (ICD-9-CM) and International Statistical Classification of Diseases, Tenth Revision, Clinical Modification (ICD 10-CM)

Variables	Definition	
	ICD-9-CM	ICD-10-CM
**Comorbidities listed in CCI**		
Asthma	493	J45
Anxiety disorder	300	F41
Bipolar disorder	296	F30, F31, F34.0, F34.8, F34.9
Cardiac arrhythmia	427	I48, I49
Cataract	366	H25, H26, H28
Coronary artery disease	410, 411, 412, 413, 414	I20, I21, I22, I23, I24, I25
Cerebrovascular disease	430, 431, 432, 433, 434, 435, 436, 437, 438	I60, I61, I62, I63, I64, I65, I66, I67, I68, I69
Chronic kidney disease	250.4, 403.01, 403.11, 403.91, 404.01, 404.02, 4040.3, 404.11, 404.12, 404.13, 404.91, 404.92, 404.93, 581, 585, 586, 593.6, 791.0	E10.2, E11.2, E13.2, I12, I130, I132, N04, N06, N18, N19, R80
Congestive heart failure	428	I50
Chronic obstructive pulmonary disease	491, 492, 494, 495, 496	J43, J44
Dementia	290, 294.0, 294.1, 331.0, 331.1, 331.2, 331.82, 331.83, 331.89, 331.9	F00, F01, F02, F03, G30, G31.0, G31.1, G31.83, G31.84, G31.9, G32
Delirium	293	F05
Diabetes mellitus	250	E10, E11, E13, E14
Epilepsy	345	G40, G41
Glaucoma	365	H40, H41, H42
Gastroesophageal reflux disease	530.2, 530.81, 531, 532, 533, 534	K21, K22.1, K25, K26, K27, K28
Hyperlipidemia	272	E78
Hypertension	401, 402, 403, 404, 405	I10, I11, I12, I13, I15
Liver disorder	571, 572, 573	K70, K71, K72, K73, K74, K75, K76, K77
Osteoporosis	733.0, 733.1	M80, M81, M82
Parkinson	332	G20, G21
Rheumatoid arthritis	714	M05, M06
Sleep disorder	780.5	G47
Schizophrenia	290.8, 290.9, 295, 301.2	F20, F21, F25, F28, F29, F32.3, F33.3
Systemic lupus erythematosus	710	M32
**Post-operative complications**		
Anastomotic leakage	997.4	K91.89
Bacteremia/sepsis	998.59+790.7, 998.59+038.9+785.59	T81.4XXA+R78.81, 81.4XXA+A41.9+R65.21
Acute myocardial infarction	997.1+410.91	I97.89+I21.3

© 2025 Chen PC et al. *JAMA Network Open*.

Deep vein thrombosis	997.2+453.8	T81.72XA+I82.409

© 2025 Chen PC et al. *JAMA Network Open*.

eTable 3. Neoadjuvant/adjuvant therapies of patients with resectable LARC after propensity score fine stratification.

Variables[a]	NRT followed by surgery (n=1,308)	Upfront surgery (n=2,484)	Standardized mean difference
Neoadjuvant radiotherapy			
Long-course	1,077 (82.3)	.	.
Short-course	231 (17.7)	.	.
Interval between NRT initiation and surgery (day), median (IQR)	86 (72-100)	.	.
Neoadjuvant chemotherapy			
5-fluorouracil-based	1,028 (78.6)	.	.
Oxaliplatin-based	68 (5.2)	.	.
Adjuvant radiotherapy	4 (0.3)	389 (15.7)	-0.59
Adjuvant chemotherapy			
5-fluorouracil-based	1,216 (93.0)	1,645 (66.2)	0.70
Oxaliplatin-based	290 (22.2)	602 (24.2)	-0.05

LARC, locally advanced rectal cancer; NRT, neoadjuvant radiotherapy

[a] Data are expressed as patient number (percentage) unless otherwise indicated.

© 2025 Chen PC et al. *JAMA Network Open*.

eTable 4. Perioperative outcomes of patients with resectable LARC after propensity score fine stratification.

Variables[a]	NRT followed by surgery (n=1,308)	Upfront surgery (n=2,484)	SMD
Operation			
Hartmann's operation	5 (0.4)	29 (1.2)	-0.09
Abdominoperineal resection	116 (8.9)	209 (8.4)	0.02
Low anterior resection	1,187 (90.7)	2,246 (90.4)	0.00
Diverting stoma creation	857 (65.5)	1,018 (41.0)	0.51
Pathology			
Pathological stage			
pT0	213 (16.3)	0 (0)	0.62
pT1	67 (5.1)	121 (4.9)	0.01
pT2	341 (26.1)	502 (20.2)	0.14
pT3	639 (48.9)	1,698 (68.4)	-0.40
pT missing	48 (3.7)	162 (6.5)	-0.13
pN0	832 (63.6)	1,318 (53.1)	0.22
pN1	338 (25.8)	619 (24.9)	0.02
pN2	130 (9.9)	543 (21.8)	-0.33
pN missing	8 (0.6)	5 (0.2)	0.07
pM1	14 (1.1)	27 (1.1)	0.00
Numbers of lymph nodes harvested, median (IQR)	14 (9-19)	20 (14-25)	-0.74
Numbers of positive lymph nodes, median (IQR)	0 (0-1)	0 (0-3)	-0.36
Surgical margin			
Involved	41 (3.1)	122 (4.9)	-0.09
Missing	13 (1.0)	33 (1.3)	-0.03
Postoperative complications at 90 days			
Anastomotic leak	99 (7.6)	210 (8.5)	-0.03
Bacteremia	111 (8.5)	199 (8.0)	0.02
Acute myocardial infarction	9 (0.7)	17 (0.7)	0.00
Deep vein thrombosis	17 (1.3)	10 (0.4)	0.10

LARC, locally advanced rectal cancer; NRT, neoadjuvant radiotherapy; SMD, standardized mean difference.

[a] Data are expressed as patient number (percentage) unless otherwise indicated.

© 2025 Chen PC et al. *JAMA Network Open*.

eTable 5. Risks of overall survival and local recurrence at 3 years following NRT followed by surgery versus upfront surgery for overall resectable LARC and according to different tumor heights estimated using multivariate Cox regression.

Variables	Hazard ratio (95% CI)[a,b]	P	P for interaction
Overall survival[d]			
Overall patients	0.73 (0.57-0.93)	0.01*	
Upper rectal cancer	1.48 (0.79-2.79)	0.22	0.01*
Middle rectal cancer	0.70 (0.48-1.03)	0.07	0.95
Lower rectal cancer	0.66 (0.45-0.96)	0.03*	Reference
Local recurrence			
Overall patients	0.74 (0.49-1.12)	0.16	
Upper rectal cancer	1.04 (0.23-4.77)	0.96	0.10
Middle rectal cancer	1.13 (0.60-2.15)	0.71	0.43
Lower rectal cancer	0.55 (0.31-0.99)	0.04*	Reference

* signifies statistical significance (P<0.05).

NRT, neoadjuvant radiotherapy; LARC, locally advanced rectal cancer

[a] Adjusted for the variables considered in the propensity score calculation.

[b] For overall survival, hazard ratio of death is presented.

© 2025 Chen PC et al. *JAMA Network Open*.

eFigure 1. Patient selection

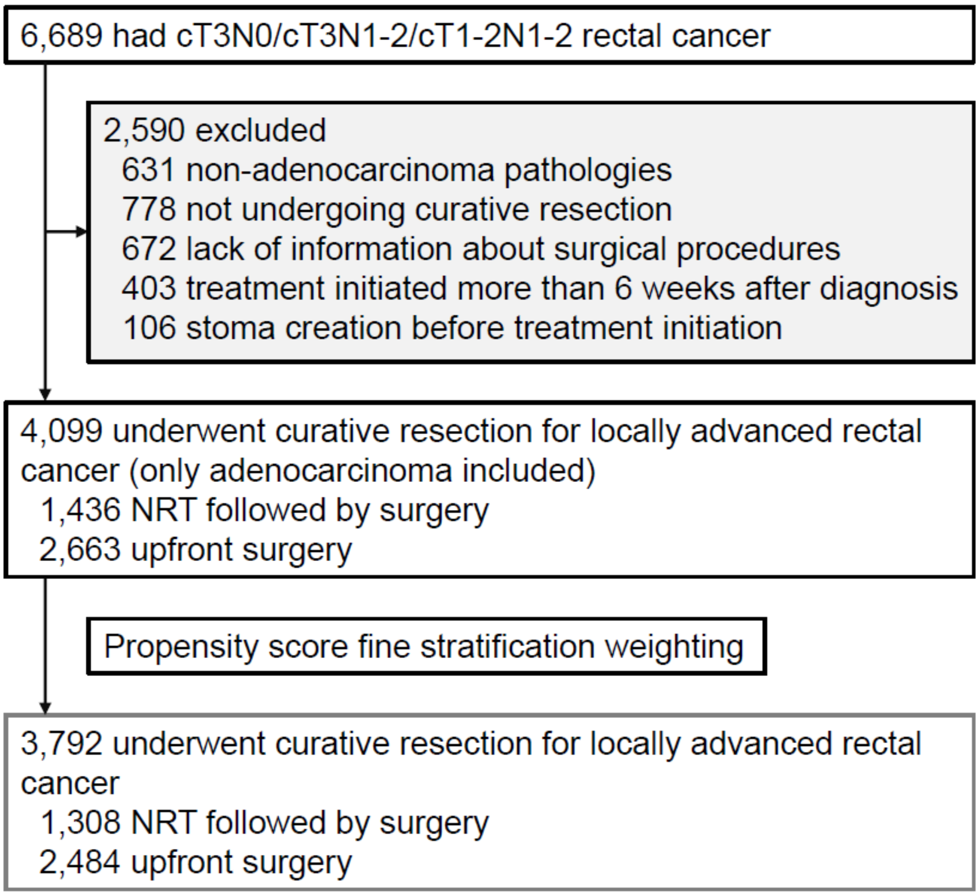

© 2025 Chen PC et al. *JAMA Network Open*.

eFigure 2. Bubble charts.

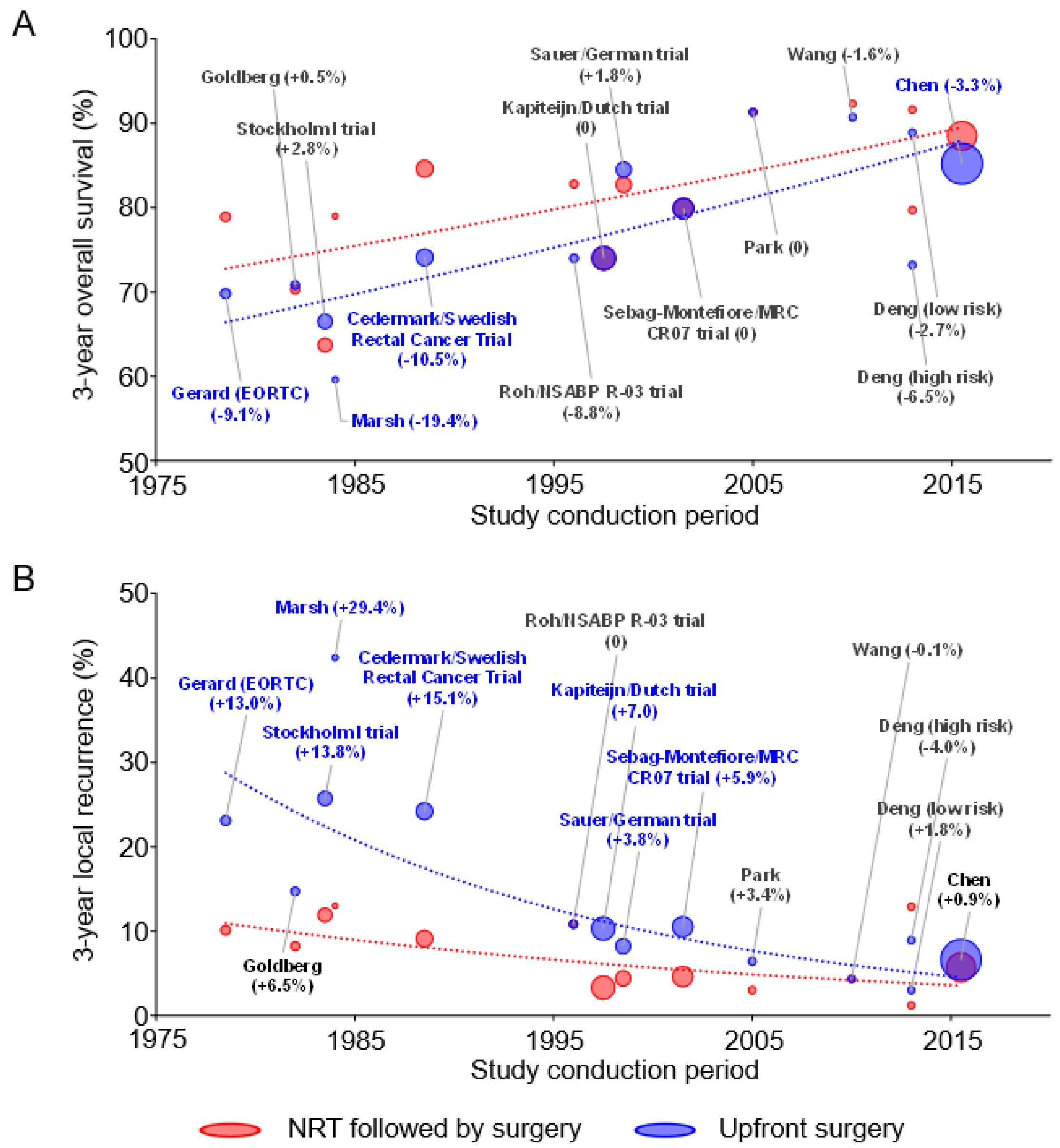

eFigure 2. Bubble charts summarizing (A) overall survival and (B) local recurrence rates at 3 years reported in the trials comparing NRT followed by surgery with upfront surgery for resectable LARC. Only outcomes following curative resection were extracted from these studies. The Stockholm II trial was omitted due to a substantial overlap in patient inclusion with the Swedish Rectal Cancer Trial. The differences between upfront surgery and NRT followed by surgery in each trial are

© 2025 Chen PC et al. *JAMA Network Open*.

specified in parentheses, and study results significantly favoring NRT followed by surgery are colored blue.

© 2025 Chen PC et al. *JAMA Network Open*.
