# Supplementary material for: Neoadjuvant Radiotherapy vs Up-Front Surgery for Resectable Locally Advanced Rectal Cancer
Source: JAMA Netw Open. 2025 May 7;8(5):e259049. doi: 10.1001/jamanetworkopen.2025.9049 (PMC12059978; doi:10.1001/jamanetworkopen.2025.9049)
Supplement: Supplement 2. — Data Sharing Statement [file jamanetwopen-e259049-s002.pdf]

Data Sharing Statement

Chen. Neoadjuvant Radiotherapy vs Up-Front Surgery for Resectable Locally Advanced Rectal Cancer. *JAMA Netw Open*. Published May 07, 2025. doi:10.1001/jamanetworkopen.2025.9049

Data
Data available: No

Additional Information
Explanation for why data not available: Individual patient data are not accessible based on the policy of national registries used in this study.
